# Anti-inflammatory Effects of Siponimod in a Mouse Model of Excitotoxicity-Induced Retinal Injury

**DOI:** 10.1007/s12035-023-03535-0

**Published:** 2023-08-05

**Authors:** Devaraj Basavarajappa, Vivek Gupta, Nitin Chitranshi, Deepa Viswanathan, Veer Gupta, Roshana Vander Wall, Viswanthram Palanivel, Mehdi Mirzaei, Yuyi You, Alexander Klistorner, Stuart L. Graham

**Affiliations:** 1 Macquarie Medical School, Faculty of Medicine, Health and Human Sciences, Macquarie University, North Ryde, Sydney, NSW 2109 Australia; 2 School of Medicine, Deakin University, Geelong, VIC Australia; 3 Save Sight Institute, The University of Sydney, Sydney, NSW 2000 Australia

**Keywords:** Excitotoxicity, NMDA, Glaucoma, Glia, Neuroinflammation, Inflammasome, Sphingosine-1-phosphate, Siponimod

## Abstract

Glaucoma is a leading cause of permanent blindness worldwide and is characterized by neurodegeneration linked to progressive retinal ganglion cell (RGC) death, axonal damage, and neuroinflammation. Glutamate excitotoxicity mediated through N-methyl-D-aspartate (NMDA) receptors plays a crucial role in glaucomatous RGC loss. Sphingosine 1-phosphate receptors (S1PRs) are important mediators of neurodegeneration and neuroinflammation in the brain and the retina. Siponimod is an immunomodulatory drug for multiple sclerosis and is a selective modulator of S1PR subtypes 1 and 5 and has been shown to have beneficial effects on the central nervous system (CNS) in degenerative conditions. Our previous study showed that mice administered orally with siponimod protected inner retinal structure and function against acute NMDA excitotoxicity. To elucidate the molecular mechanisms behind these protective effects, we investigated the inflammatory pathways affected by siponimod treatment in NMDA excitotoxicity model. NMDA excitotoxicity resulted in the activation of glial cells coupled with upregulation of the inflammatory NF-kB pathway and increased expression of TNFα, IL1-β, and IL-6. Siponimod treatment significantly reduced glial activation and suppressed the pro-inflammatory pathways. Furthermore, NMDA-induced activation of NLRP3 inflammasome and upregulation of neurotoxic inducible nitric oxide synthase (iNOS) were significantly diminished with siponimod treatment. Our data demonstrated that siponimod induces anti-inflammatory effects via suppression of glial activation and inflammatory singling pathways that could protect the retina against acute excitotoxicity conditions. These findings provide insights into the anti-inflammatory effects of siponimod in the CNS and suggest a potential therapeutic strategy for neuroinflammatory conditions.

## Introduction

Glaucoma is one of the leading causes of irreversible blindness globally and is projected to affect over 100 million people worldwide by 2040 [1, 2]. It is a multifactorial complex neurodegenerative disease characterized by progressive loss of retinal ganglion cells (RGCs) and optic nerve degeneration. Elevated intraocular pressure (IOP) is considered as an important risk factor for glaucoma pathogenesis [3–5]. However, many glaucoma patients never manifest raised IOP, and glaucoma progression is observed despite lowering IOP [2]. Hence, it is important to understand underlying IOP-independent glaucomatous degenerative mechanisms and investigate the potential therapeutic targets that could suppress degenerative pathways. Immune response to neuronal injuries and chronic neuroinflammatory processes are well implicated in glaucomatous degeneration and disease progression, both in human and experimental glaucoma [6, 7]. The early response of resident immune cells involving the activation of microglia and macroglia (astrocytes and Muller cells) is observed prior to RGC degeneration in experimental glaucoma injury [8, 9]. Upregulation of inflammasome cascade, the release of a variety of pro-inflammatory cytokines, and neurotoxic molecules in neuroinflammatory responses are deleterious to neuronal tissue and exacerbate glaucomatous degeneration [10–12].

Glutamate is an abundant excitatory neurotransmitter in the central nervous system (CNS), and its overproduction leads to neuronal cell death in several important neurodegenerative diseases including glaucoma [13–15]. In the retina, glutamate excitotoxicity is a significant component in glaucomatous degeneration and is mediated particularly through N-methyl-D-aspartate (NMDA) receptors [16, 17]. Excessive activation of NMDA receptors plays a substantial role in neuronal cell death via the influx of calcium ions (Ca^2+^) into the cells which in turn leads to mitochondrial damage, oxidative stress, and activation of several calcium-dependent pathways in neurons [17, 18]. In rodent models, intravitreal injection of NMDA is commonly used to study excitotoxicity-induced RGC death [19–21]. NMDA-mediated neuronal injury triggers the activation of retinal glial cells and subsequent upregulation of inflammasome, pro-inflammatory cytokines, and chemokines in the neuroinflammatory process adversely affecting the retinal structure and function [21–23]. In this regard, the suppression of proinflammatory pathways is a promising protective strategy to protect RGC degeneration.

Sphingosine 1-phosphate (S1P) and its receptors are well expressed in the CNS, including the retina, and regulate several pathophysiological processes. S1P binds to five G-protein-coupled receptors, termed S1PR1-5. S1P receptor signaling regulates several cellular functions including cell survival, differentiation, migration, angiogenesis, and immune cell recruitment[24–26]. S1PR1 is the most widely expressed S1P receptor subtype that mediates cellular survival and neuroinflammation in the CNS[27]. Siponimod (BAF312) is a US Food and Drug Administration (FDA)-approved immunosuppressant drug for the management of secondary multiple sclerosis (MS). It is an S1P analogue that selectively modulates the S1P1 and S1P5 receptors. Modulation of S1PR1 in the peripheral lymphocytes by siponimod impairs their egress from lymph nodes and thus migration into the CNS and reduces inflammation [28, 29]. Because of its lipophilic nature, siponimod crosses the blood-brain barrier (BBB) and can directly interact with the CNS-resident cells expressing S1P receptors. In several preclinical models, siponimod has been shown to be neuroprotective through its survival signaling and anti-inflammatory effects [30–32]. We previously showed that siponimod treatment exerted neuroprotection against experimental glaucoma injury and further protected retinal structure and function against acute NMDA excitotoxicity [33]. Here, we aimed to understand the mechanism of protective effects by investigating the anti-inflammatory effects of siponimod against NMDA ecotoxicity, evaluating the glial activation, nuclear factor kappa B (NF-kB) pathway, nuclear receptor expression (NR4A), cytokine expression, and nucleotide-binding leucine-rich repeat-containing receptor family, pyrin domain containing 3 (NLRP3) inflammasome cascade.

## Materials and Methods

### Experimental Animals

Wild-type C57BL/6 mice (4–6 weeks) were obtained from the Animal Resources Centre, Perth, Australia, and housed at the Animal Care Facility of Macquarie University under specific pathogen-free conditions. All animal experiments in this study were conducted in accordance with the Australian Code of Practice for the Care and Use of Animals for Scientific Purposes and the guidelines of the ARVO (the Association for Research in Vision and Ophthalmology) Statement for the Use of Animals in Ophthalmic and Vision Research, and all procedures were approved by Animal Ethics Committee of Macquarie University. The animals were maintained under standard laboratory conditions with 12-h light/dark cycles at a controlled temperature (21 to 28 °C) in an air-conditioned room. Siponimod (BAF312) (Novartis, Basel, Switzerland) was administered orally to the recipient mice group through a diet (10 mg/kg of diet) as described previously [33]. Each cage had 3–4 mice with full access to pellet food and water ad libitum.

### Intravitreal NMDA Injection

Intravitreal injections were performed under an operating surgical microscope (Carl Zeiss, Germany) using the procedure explained in our previous studies [33, 34]. Briefly, mice were anesthetized by placing them in an induction chamber supplied with 2–5% isoflurane in oxygen, and then, the animals were maintained on 1–3% isoflurane in oxygen (0.6–1 L/min flow of oxygen) on a heating pad during the injection procedure. The pupils were dilated with 1% tropicamide solution and a single intravitreal injection of NMDA solution (Sigma-Aldrich, St. Louis, MO, USA) in 2 μL volume (30 nmol per eye) was injected into the vitreous chamber behind the lens using a Hamilton syringe connected to a disposable 33G needle (TSK Laboratory, Tochigi, Japan). In the case of the control animal group, the mice eyes received an equivalent volume of sterile PBS. Seven days after the NMDA injection, the animals were sacrificed to harvest the tissues for analysis.

### Immunofluorescence Staining

Immunofluorescence staining of the retinal and optic nerve cryosections was performed as described previously [35–37]. The tissues were harvested from the mice, transcardially perfused with PBS and freshly prepared 4% paraformaldehyde (PFA). To maintain the same orientation during tissue embedding, the eyeballs were marked with tissue marking dye. The harvested eyes were then fixed for 1 h in 4% PFA and washed three times with PBS. Post-fixed eyes were immersed in 30% sucrose solution overnight before tissue embedding in optimal cutting temperature compound (OCT) (Sakura Finetek, CA, USA) on dry ice. Seven-micrometer-thick sagittal retinal sections or 5-μm optic nerve cross-sections were treated with a blocking solution containing 0.3% Triton X-100 and 5% donkey serum (Sigma-Aldrich, St. Louis, MO, USA) in PBS for 2 h at room temperature. After blocking, the sections were incubated with primary antibodies in antibody dilution buffer (1 × PBS/2% BSA/0.3% Triton X-100) overnight at 4 °C. The following primary antibodies and their dilution were used for immunofluorescence staining: rabbit anti-Iba1 (Ionized calcium-binding adaptor molecule 1) (microglial marker; 1:1500; 019-19741, FUJIFILM Wako Shibayagi); rabbit anti-GFAP (glial fibrillary acidic protein) (a marker for reactive gliosis; 1:1500; Z0334, Agilent, Santa Clara, CA, USA); NF-κB p65 (1:1000, (D14E12) Cell Signaling Technology, Cat# 8242), rabbit anti-Nur77 (1:1000, Abcam, Cat# ab283264), mouse anti-Iba1 (1:1000, Abcam, Cat# ab283319), mouse anti-GFAP (1:1000, (GA5) Cell Signaling Technology, Cat# 3670) and iNOS (1:1000, (D6B6S) Cell Signaling Technology, Cat# 13120). Tissue sections were then washed three times with PBS and incubated with appropriate secondary antibodies (1;1000, donkey anti-rabbit Cy3 or donkey anti-mouse Alexa Fluor 488; Jackson ImmunoResearch Labs) for 60 min at room temperature. After washing the sections with PBS, the slides were mounted with anti-fade mounting media with Prolong DAPI (Life Technologies, Eugene, OR, USA). Acquisition of images was performed using a Zeiss fluorescence microscope (ZEISS Axio Imager, Carl Zeiss, Oberkochen, Germany). All quantifications of relative fluorescence intensity were obtained from the images of three sections of each animal tissue using the ImageJ software (version 1.52; NIH, Bethesda, MD, USA)[38, 39].

### Western Blot Analysis

Retinal tissues were isolated from eyes and span frozen in liquid nitrogen before being resuspended in ice-cold lysis buffer (20 mM Tris-HCl, pH 8.0, 1% Triton X-100, 2 mM EDTA, 2 mM PMSF, 100 mM NaCl, 1 mM Na_3_VO_4_, 0.1% SDS, and complete protease inhibitor cocktail). Retinae were homogenized by sonication on ice and centrifuged at 10,000 × g for 10 min at 4 °C to collect protein supernatants. Retrieved supernatant protein concentrations were determined by Micro BCA assay (Thermo Fisher Scientific, USA). Retinal proteins (20 µg each sample) were resolved by SDS-PAGE and transferred to nitrocellulose membrane by electroblotting (Invitrogen iBlot2, Thermo Fisher Scientific, USA) as described previously [40–42]. The nitrocellulose membranes were washed with TTBS (20 mM Tris-HCl pH 7.4, 100 mM NaCl, and 0.1% Tween 20) before blocking with BSA (3%) in TTBS for 1 h at room temperature. The blot membranes were then incubated with primary antibodies at 4 °C overnight. The primary antibodies with concentration used were rabbit polyclonal anti-Iba1 (1:1000; FUJIFILM Wako Shibayagi, 019-19741), rabbit polyclonal anti-GFAP (1:1500; Agilent, Z0334), rabbit monoclonal NF-κB p65 (1:1000, (D14E12) Cell Signaling Technology, Cat# 8242), rabbit monoclonal NLRP3 (1:1000, (D4D8T) Cell Signaling Technology, Cat# 15101), TNF-α (1:1000, (D14E12) Cell Signaling Technology, Cat# 2022), rabbit monoclonal iNOS (1:1000, (D6B6S) Cell Signaling Technology, Cat# 13120), rabbit polyclonal IL1β (1:1000, Abcam, ab234437), rabbit polyclonal IL-6 (1:1000, Abcam, Cat# ab6672), rabbit monoclonal Caspase-1 (1:1000, Abcam, Cat# ab207802), Nur77 (1:1000, Abcam, Cat# ab283264), Nurr1(1:1000, Abcam, Cat# ab41917), and anti-β-actin (1:5000; Abcam, Cat# ab6276). After primary antibody incubations, the membranes were washed four times (5 min each) with TTBS and incubated with a secondary antibody conjugated to horseradish peroxidase (anti-rabbit 1:5000, and anti-mouse 1:5000, Jackson ImmunoResearch Labs) for 1 h at room temperature. The blot membranes were then washed four times and chemiluminescence bands captured in a Bio-Rad ChemiDoc^MP^ Imaging system (Bio-Rad Laboratories, Inc., CA, USA) using enhanced chemiluminescence substrate (Super Signal West Femto Maximum Sensitive Substrate; Thermo Fisher Scientific) according to the manufacturer’s instructions. The analysis of protein band intensities was performed with the ImageJ software.

### Statistical Analysis

All statistical analysis in this study was performed using the GraphPad Prism 8 software (GraphPad Software Inc., San Diego, CA, USA). The retinal and optic nerve tissues from the experimental groups were utilized for immunofluorescence and western blot analysis of target proteins. The number of samples (*n*) in each figure represents the tissues from the different animals of the same group. Statistical analysis between the groups was performed by one-way ANOVA followed by Tukey’s test for multiple comparisons (GraphPad Prism). Error bars are presented as mean ± standard deviation of the mean (SD) for given n sizes and a *P*-value below 0.05 was considered statistically significant.

## Results

### Siponimod Treatment Attenuated Glial Activation in NMDA Excitotoxicity

**Fig. 1:**
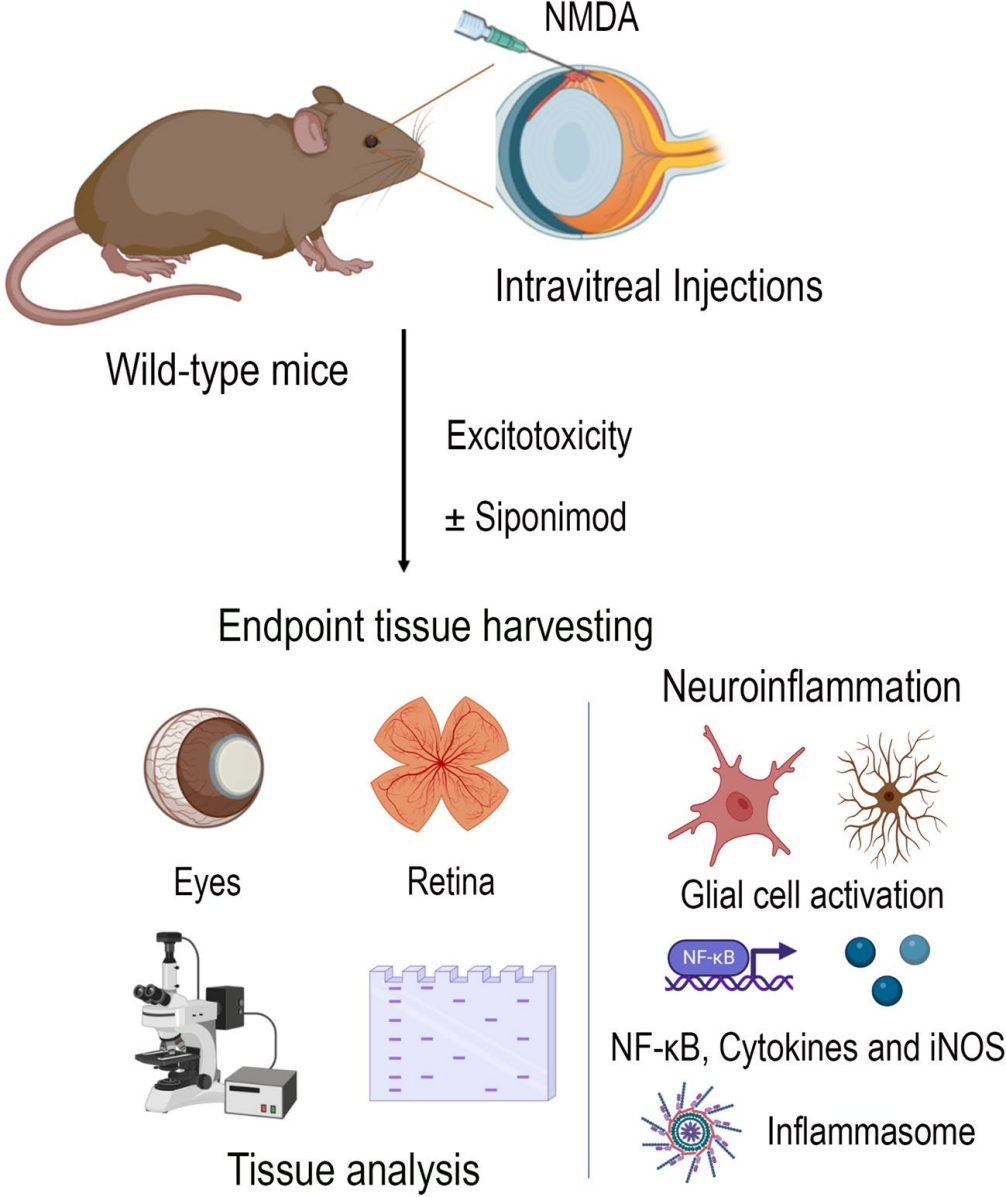
Schematic figure depicting the experimental design and analysis of retinal tissue in NMDA excitotoxicity model. Mice (C57BL/6) received a single intravitreal injection of NMDA, and siponimod was administered through diet. The harvested tissues after 7 days of injections were examined for neuroinflammatory changes by analyzing glial cell activation, NF-kB pathway, cytokines, and iNOS expression, and inflammasome activation to understand the anti-inflammatory effects of siponimod during retinal NMDA excitotoxicity

**Fig. 2:**
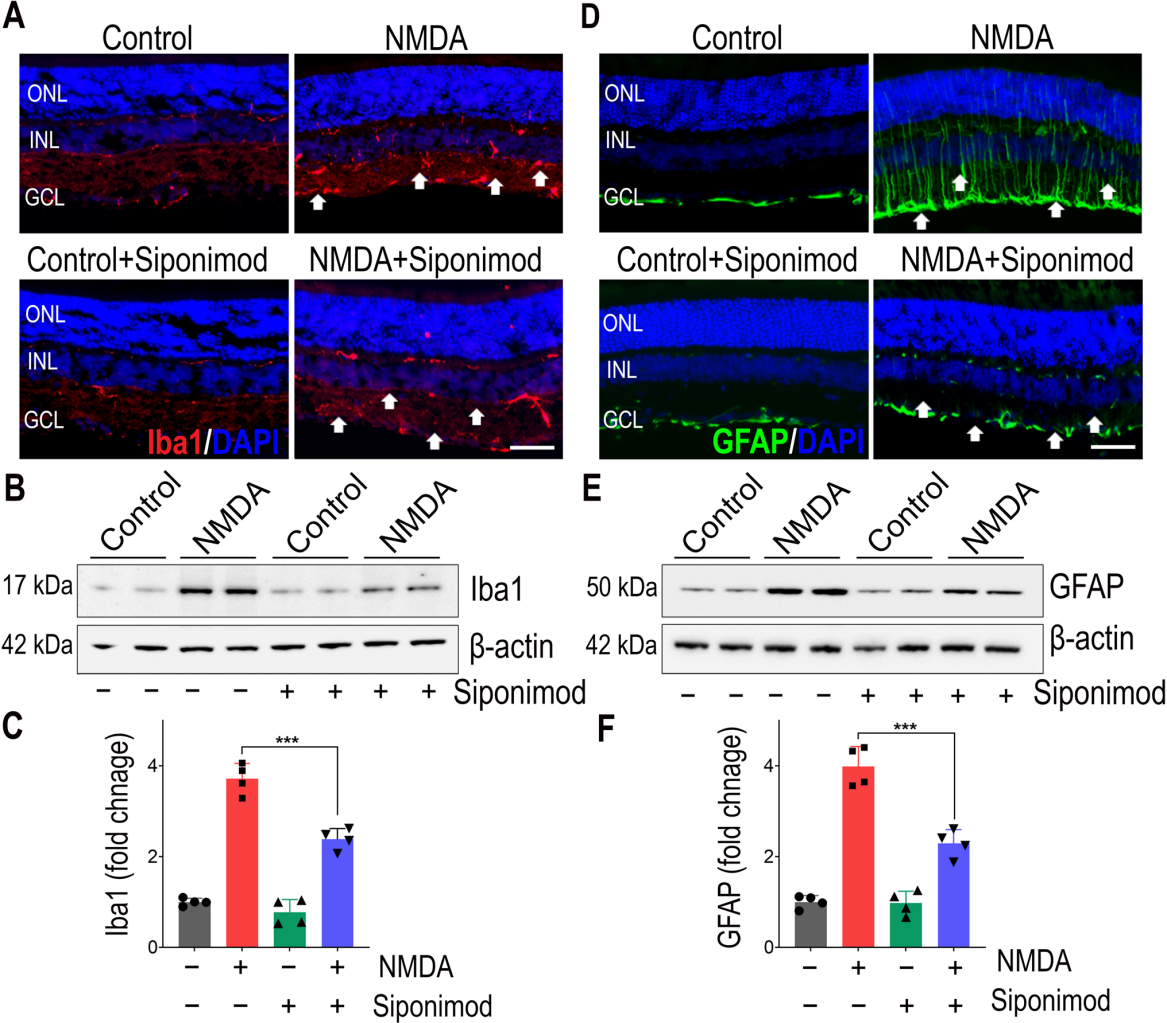
Siponimod treatment reduces retinal glial activation in NMDA excitotoxicity. **A** Immunofluorescence images of the eye sections stained with Iba1 (red) for microglia and DAPI (blue) (representative images, scale bar: 50 µm; ONL, outer nuclear layer; INL, inner nuclear layer; GCL, ganglion cell layer) showing the microglial activation and its modulation with siponimod treatment in NMDA treated retinas (arrows indicate the changes in Iba1 immunoreactivity). **B** Western blot analysis of retinal tissues for Iba1 levels following NMDA excitotoxicity (representative blots). **C** Densitometric quantitative analysis of Iba1 blot densities (normalized to β-actin) showing the increased levels of Iba1 in NMDA treated retinas and its significant reduction with siponiomd treatment (***P < 0.001 one-way ANOVA analysis with Tukey’s multiple comparisons test, *n* = 4 per group). **D** Immunofluorescence images of the eye sections stained with GFAP (green) and DAPI (blue) for analysis of reactive gliosis (Muller glia) (representative images, Scale bar: 50 µm; arrows indicate the changes in GFAP and hypertrophy). **E** Western blot analysis for GFAP protein expressions in retinas lysates (representative blots) and **F** their densitometric quantitative analysis showing the significantly reduced expression of GFAP with siponimod treatment during NMDA excitotoxicity (***P < 0.001, one-way ANOVA analysis with Tukey’s multiple comparisons test *n* = 4 per group)

**Fig. 3:**
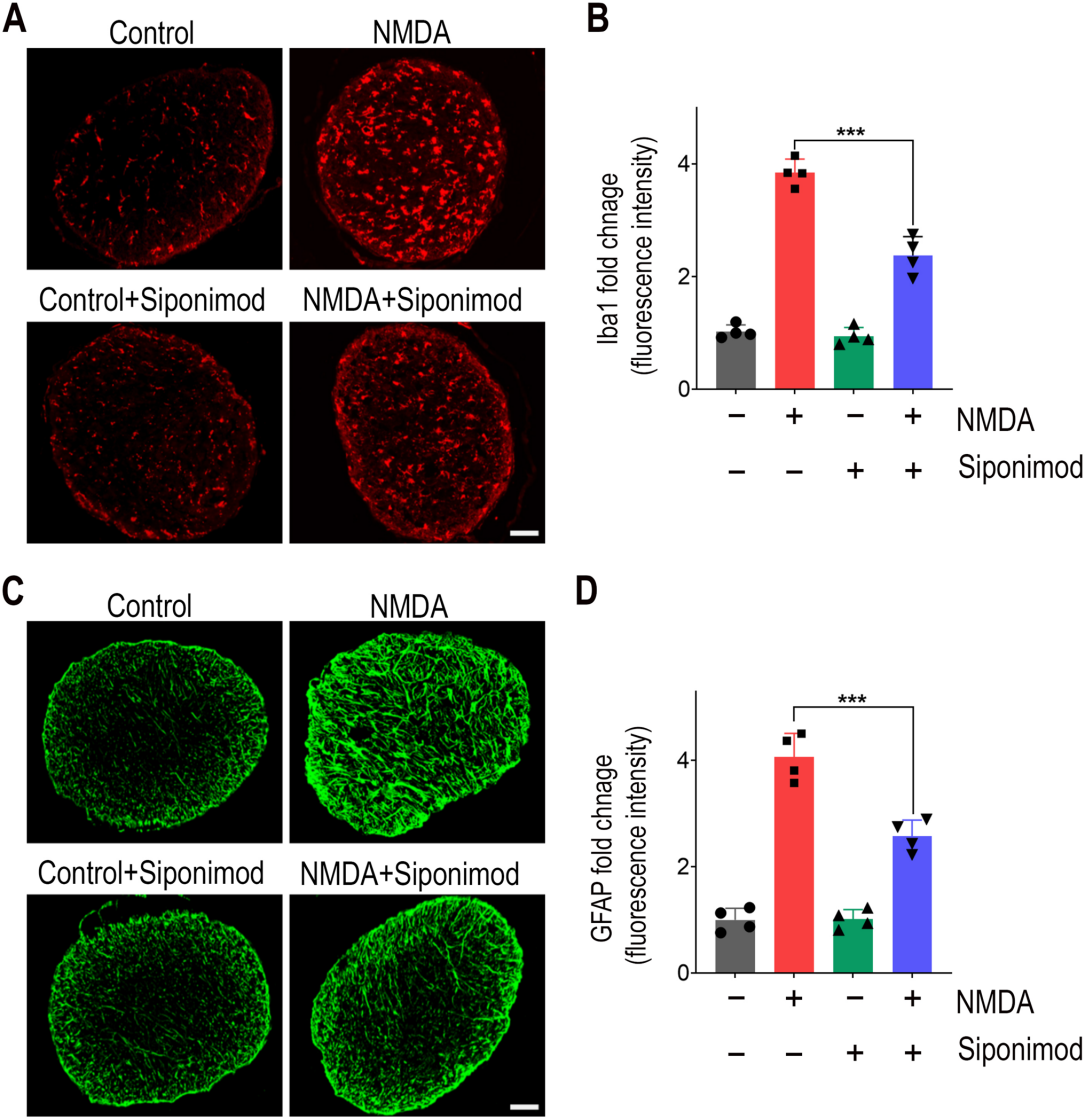
Siponimod treatment attenuates glial activation in optic nerve in NMDA excitotoxicity. **A** Representative immunofluorescence images of optic nerve cross-sections (scale bar: 50 µm) stained for microglia with Iba1 (red) and **B** quantification of Iba1 immunoreactivity showing NDMA excitotoxity-induced microglia activation was significantly reduced with siponiomd treatment (***P < 0.001, one-way ANOVA analysis with Tukey’s test, *n* = 4 optic nerves per group). **C** Immunofluorescence images of optic nerve cross-sections stained with GFAP stained (green) for analysis of reactive astrogliosis (representative images, scale bar: 50 µm) and **D** their quantitative immunoreactivity measurements showing the significant suppressive effects of siponimod treatment on astrogliosis during NMDA excitotoxicity (***P < 0.001, one-way ANOVA analysis with Tukey’s test, *n* = 4 per group)

The protective in vivo anti-inflammatory effects of siponimod on the retina were evaluated in adult C57BL/6 mice subjected to acute NMDA excitotoxicity, and the siponimod drug was administered through diet (Fig. 1). Retinal tissues were harvested from the mice eyes after 7 days of a single intravitreal injection of NMDA to evaluate the changes in the inflammatory response. Neuroinflammatory glial activation in response to NMDA excitotoxicity plays a vital role in retinal neurodegeneration [9, 22]. We evaluated the glial activation in the retinal and optic nerve tissues from the mice eyes using the markers, Iba1 for microglia, and GFAP for Müller glia and astrocytes. Immunofluorescence staining of retinal sections revealed increased Iba1 positive cells with activated morphology in response to NMDA excitotoxicity compared to the control animals (Fig. 2A), and this activation was reduced with siponimod treatment. Western blot analysis of retina lysates showed a 3.71 ± 0.33-fold increase in Iba1 expression in NDMA treated group compared to the control group. Mice treated with siponimod demonstrated significantly reduced (*P* < 0.001) Iba1 expression compared to the untreated NMDA group (Fig. 2B, C). Similar to the retina, microglial activation in response to acute NMDA excitotoxicity was observed in optic nerves. Quantitative Iba1 immunoreactivity measurements showed significantly reduced microglial activation (*P* < 0.001) with siponimod treatment in NMDA excitotoxicity (Fig. 3A, B).

We next evaluated reactive gliosis by assessing the expression of intermediate filament protein GFAP. In control animals, the immunoreactivity of GFAP was confined to the GCL in the retinal cross-sections. NMDA-treated retinas showed intensified GFAP labeling with an extended Müller cell process into the inner nuclear layer (INL) (Fig. 2D). Similarly, in the NMDA-treated mice, hypertrophic reactive astrogliosis with GFAP upregulation was observed in optic nerve sections (Fig. 3C). Mice treated with siponimod showed diminished reactive gliosis both in the retain and optic nerves. Western blotting quantitative analysis of retinal lysates for GFAP expression showed 3.98 ± 0.44-fold upregulation in NMDA treatment compared to the control mice group. Siponimod treatment significantly suppressed GFAP expression compared to the untreated mice in NMDA excitotoxicity conditions (Fig. 2E, F). Furthermore, increased GFAP immunoreactivity observed in the optic nerves with NMDA excitotoxicity was reduced significantly (*P* < 0.001) in siponimod-treated mice (Fig. 3C, D). Taken together, these results suggest that the siponimod treatment contributes to the suppression of glial activation in response to acute NMDA excitotoxicity.

### Siponimod Treatment Downregulated NF-κB Activation in NMDA Excitotoxicity

**Fig. 4:**
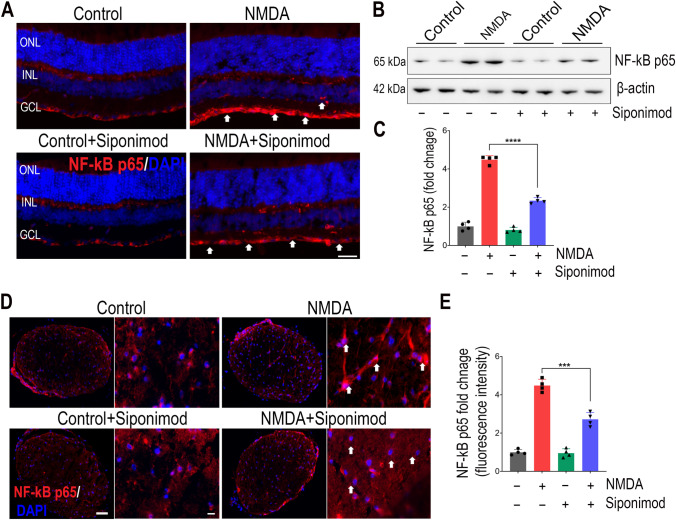
Suppression of NF-κB activation with siponimod treatment during NMDA excitotoxicity. **A**)Immunofluorescence images of the eye sections stained with NF-κB p65 (red) and DAPI (blue) (representative images, scale bar: 50 µm; ONL, outer nuclear layer; INL, inner nuclear layer; GCL, ganglion cell layer) showing the upregulation of NF-κB p65 and its modulation with siponimod treatment in NMDA treated retinas (arrows indicate the increase in NF-κB p65 immunoreactivity in NMDA excitotoxicity compared to controls and its decrease with siponimod treatment). **B** Western blot analysis of retinal tissues for the expression of NF-κB p65 following NMDA excitotoxicity (representative blots). **C** Densitometric quantitative analysis of blot densities (normalized to β-actin) showing significant suppressive effects of siponimod on NF-κB p65 upregulation in NMDA treated retinas (****P < 0.0001 one-way ANOVA analysis with Tukey’s multiple comparisons test, *n* = 4 per group). **D** Immunofluorescence images of optic nerve cross sections stained with NF-κB p65 (red) and DAPI (blue) (representative images: scale bar, 50 µm; arrows indicate the changes in NF-κB p65 immunoreactivity in NMDA excitotoxicity compared to controls and its decrease with siponimod treatment). **E** Magnified images (representative images: scale bar: 10 µm) used for quantitative immunoreactivity measurements showing the significant suppressive effects of siponimod treatment on NF-κB p65 upregulation during NMDA excitotoxicity (***P < 0.001, one-way ANOVA analysis with Tukey’s multiple comparisons test *n* = 4 per group)

Nuclear factor-kappa B (NF-κB) is a crucial transcriptional regulator of neuroinflammation, and the pro-inflammatory and neurotoxic mediators formed through NF-κB-activated inflammatory signaling have been suggested to exacerbate neuronal injury in many degenerative conditions [43, 44]. We examined the NF-κB activation by analyzing retinal NF-κB p65 expression levels. Immunofluorescence staining of retinal sections exhibited increased immunoreactivity for NF-κB p65 in the GCL and INL in the NMDA-treated group compared to the control mice. The animals treated with siponimod markedly reduced the NF-κB p65 immunoreactivity (Fig. 4A). Immunoblot densitometric analysis of retinal lysates revealed higher expression of NF-κB p65 (4.48 ± 0.21-fold increase) in NMDA-treated mice compared to the control group. This increased expression of NF-κB p65 in the NMDA-treated group was significantly reduced (*P* < 0.0001) with siponimod treatment, consistent with the immunofluorescence results (Fig. 4B, C). Similarly, increased immunoreactivity for NF-κB p65 observed in optic nerve cross-sections was significantly reduced (*P* < 0.001) with siponimod treatment (Fig. 4D, E). Combined, these results indicate that siponimod treatment downregulates the pro-inflammatory NF-κB activation in NMDA-induced inflammatory response.

### Siponimod Inhibited NMDA-Triggered NLRP3 Inflammasome Activation

**Fig. 5:**
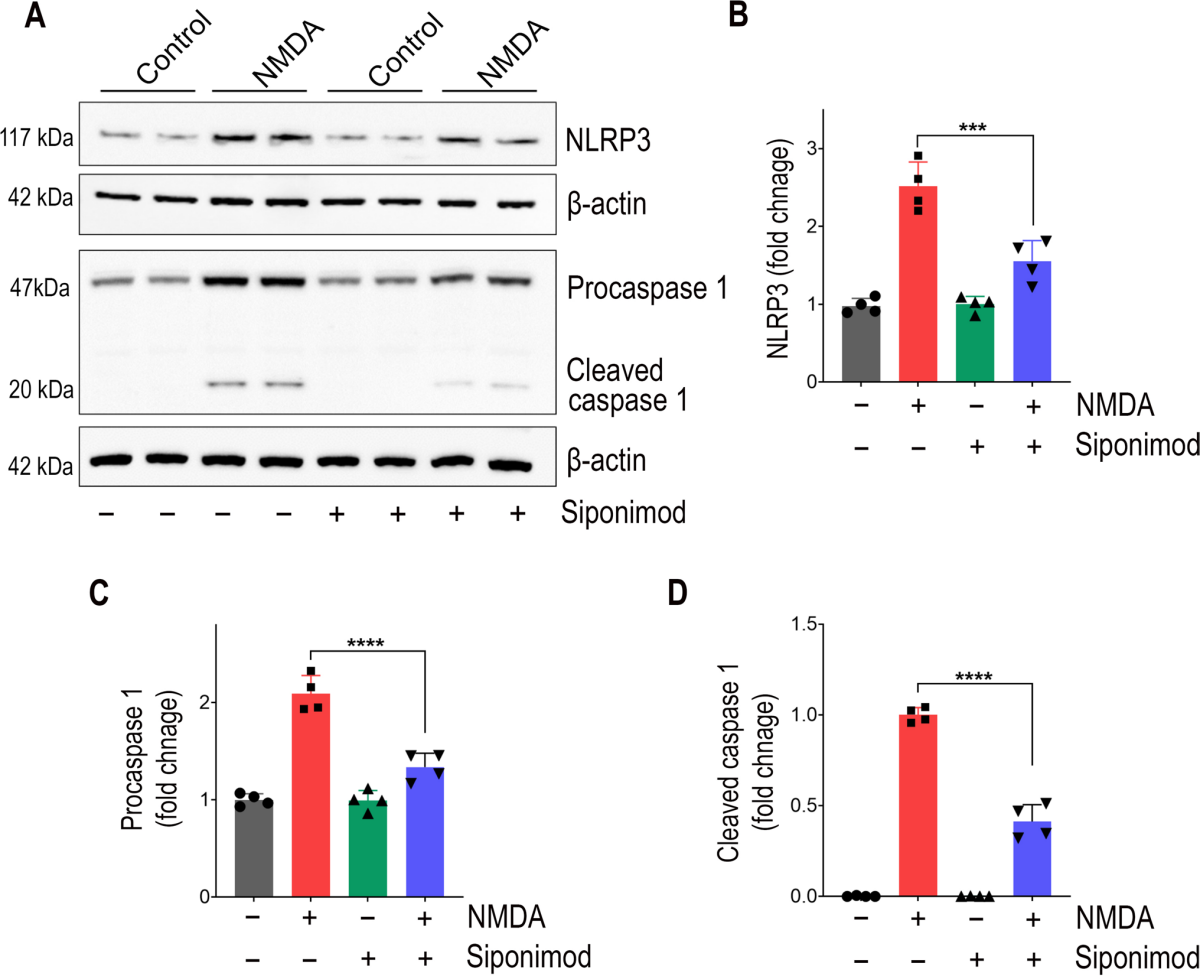
Siponimod exerts suppressive effects on NLRP3 inflammasome activation in retinal NMDA excitotoxicity. The NLRP3 inflammasome activation and its modulation by siponimod treatment was evaluated by western blotting for the expression of NLRP3, procaspase-1, and cleaved caspase-1 in the retinal tissues following NMDA excitotoxicity. **A** Representative immunoblot bands showing the expression levels of NLRP3, procaspase-1, and cleaved caspase-1 in retinal tissues. **B** Densitometric quantification of NLRP3 western blot band intensities after normalizing to β-actin. **C** Densitometric quantitative analysis for the expression levels of procaspase-1 and **D** cleaved caspase-1 (normalized to β-actin) showing the significant suppressive effects of siponimod treatment on NLRP3 inflammasome activation during NMDA excitotoxicity (***P < 0.001, ****P < 0.0001, one-way ANOVA analysis with Tukey’s multiple comparisons test, *n* = 4 per group)

NLRP3 inflammasome, a crucial driver of inflammation, is a multiprotein molecular complex implicated in several neuroinflammatory diseases including glaucoma [45, 46]. This complex consists of a central NLRP3 protein, an adaptor protein ASC (apoptosis speck-like protein), and procaspase-1. Upon activation, cleaved caspase-1 induces maturation and production of pro-inflammatory cytokines [47]. We detected the expression changes of NLRP3 inflammasome in retinal lysates by western blotting. Retinal protein levels of NLRP3, procaspase-1, and cleaved caspase-1 were markedly elevated in NMDA excitotoxicity conditions (Fig. 5A). Densitometric quantification of western blot bands revealed 2.51 ± 0.31-fold upregulation for NLRP3 and 2.10 ± 0.18-fold upregulation for procaspase-1 in the NMDA-treated retinas compared to the control group (Fig. 5B, C). Mice administered with siponimod showed significantly downregulated levels of inflammasome proteins in NMDA-treated retinas. Cleaved caspase-1 levels were significantly reduced by 58.81 ± 9.25% (*P* < 0.0001) with siponimod treatment compared to the untreated mice group in NMDA excitotoxicity conditions (Fig. 5D). This data indicates that siponimod treatment can induce anti-inflammatory effects and promotes downregulation of retinal inflammasome activation in NMDA excitotoxicity.

### Siponimod Suppressed Pro-inflammatory Cytokine Expression in NMDA-Treated Retinas

**Fig. 6:**
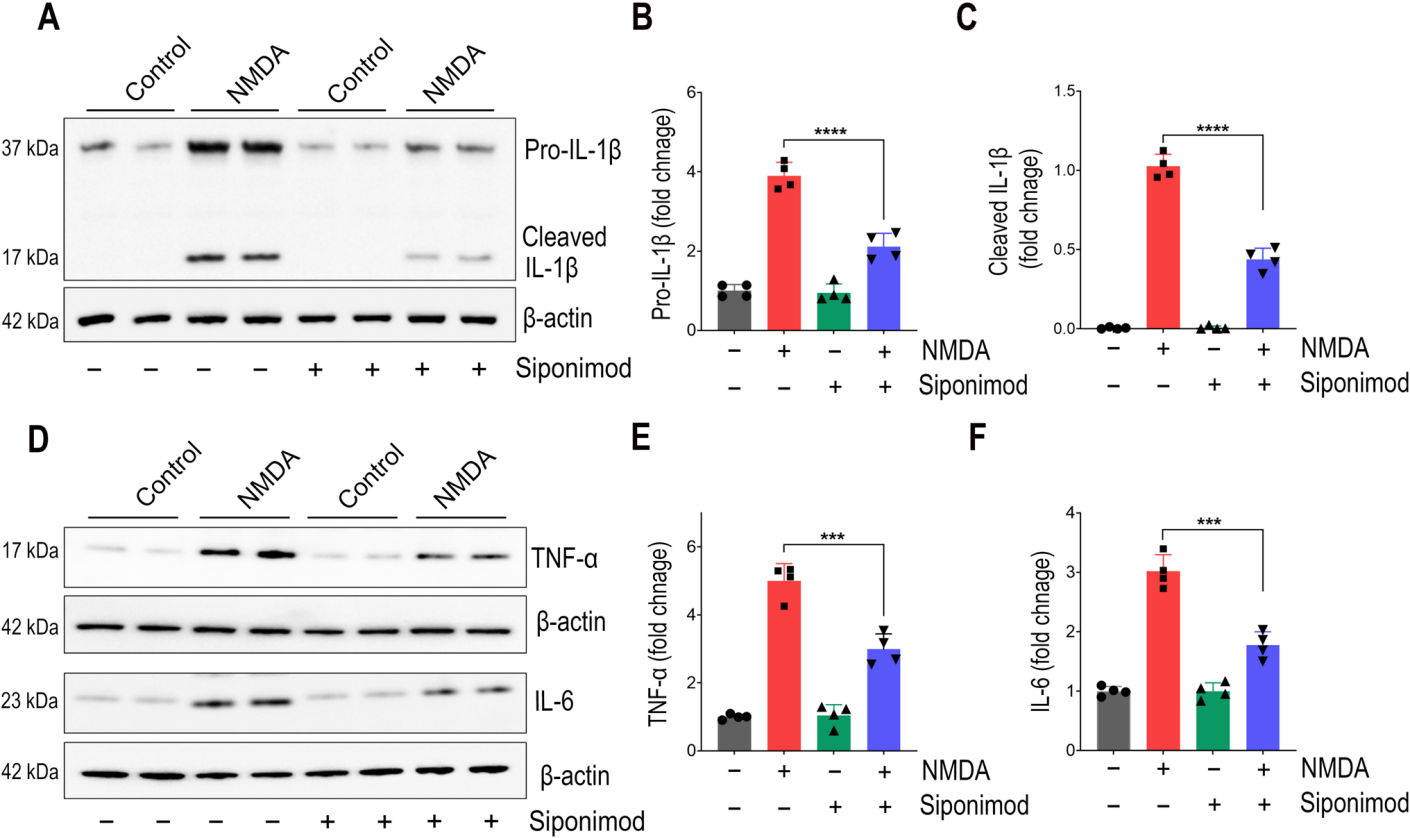
Siponimod treatment inhibits the expression of pro-inflammatory cytokines in retinal NMDA excitotoxicity. Effects of siponimod treatment on protein expressions of pro-inflammatory cytokines-IL-1β, TNF-α, and IL-6 were evaluated by western blot analysis of retinal tissues following NMDA excitotoxicity. **A** Representative immunoblot bands showing the expression levels of pro-IL-1β, and matured IL-1β in retinal tissues. **B** Densitometric quantification of pro-IL-1β and **C** matured IL-1β western blot band intensities (after normalizing to β-actin) showing the significantly reduced levels with siponimod treatment in retinal NMDA excitotoxicity that correlated with caspase-1 activation in NLRP3 inflammasome. **D** Immunoblot analysis for the expression levels of TNF-α and IL-6 (representative blots) in the retinal tissues. **E** Densitometric quantitative analysis of TNF-α and **F** IL-6 band intensities (normalized to β-actin) showing the significant suppressive effects of siponimod treatment on cytokines expression during NMDA excitotoxicity (***P < 0.001, ****P < 0.0001, one-way ANOVA analysis with Tukey’s multiple comparisons test, *n* = 4 per group)

We next evaluated crucial pro-inflammatory cytokines involved in retinal neuroinflammation such as interleukin 1β (IL-1β), tumor necrosis factor α (TNF-α), and interleukin 6 (IL-6) by western blot analysis, and these genes are transcriptional targets for NF-κB activation [48]. The activated NLRP3 inflammasome results in mature caspase-1 formation which in turn leads to IL-1β processing [47]. In the NMDA-treated retinas, the expression of pro-IL-1β was increased by 3.91 ± 0.33-fold compared to the control mice group and this upregulation was significantly reduced (*P* < 0.0001) with siponimod treatment. Furthermore, markedly increased levels of mature IL-1β were observed in the NMDA-treated retinas and significantly diminished in response to siponimod treatment (*P* < 0.0001) (Fig. 6A–C) and these results were concomitant with NLRP3 inflammasome activation. We next observed increased expression of TNF-α and IL-6 in NMDA-treated retinas compared to the control group (Fig. 6D). Densitometric quantification of western blot bands showed 5.01 ± 0.55-fold increase for TNF-α and 3.02 ± 0.28-fold increase for IL-6. In the siponimod-treated mice retinas, this upregulation was significantly suppressed (*P* < 0.001) compared to the untreated animals subjected to NMDA excitotoxicity (Fig. 6E, F). These results suggest that siponimod induces anti-inflammatory effects and suppresses expression of pro-inflammatory cytokines along with NF-κB downregulation in retinal NMDA excitotoxicity.

### Siponimod Hampered NMDA-Induced Upregulation of iNOS Expression

**Fig. 7:**
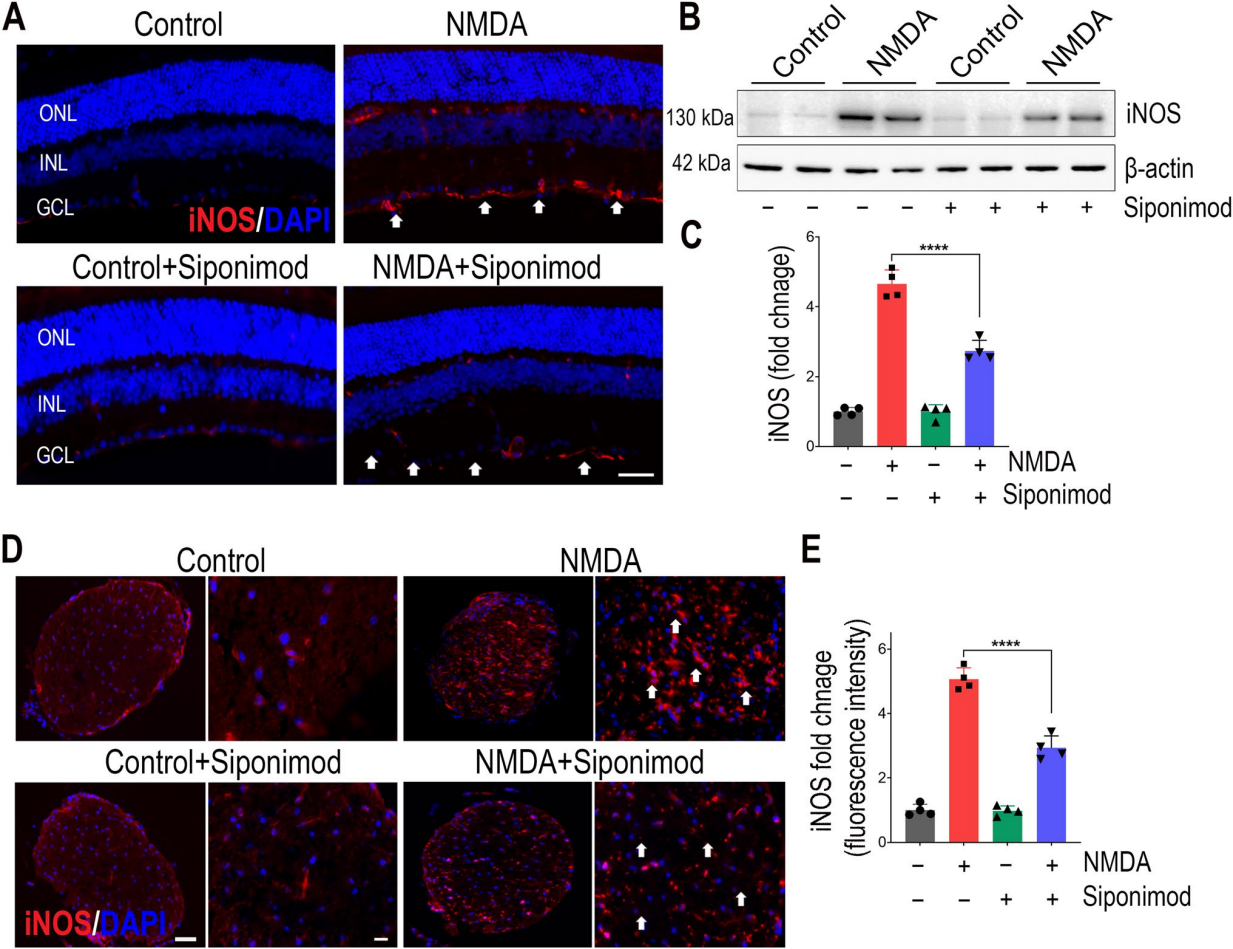
Suppressive effects of siponimod treatment on iNOS expression in retinal NMDA excitotoxicity. **A** Immunofluorescence images of the eye sections stained with iNOS (red) and DAPI (blue) (representative images: scale bar, 50 µm; ONL, outer nuclear layer; INL, inner nuclear layer; GCL, ganglion cell layer) showing the upregulation of iNOS and its modulation with siponimod treatment in NMDA treated retinas (arrows indicate the changes in iNOS immunoreactivity in NMDA excitotoxicity compared to controls and its decrease with siponimod treatment). **B** Western blot analysis of retinal tissues for the expression of iNOS following NMDA excitotoxicity (representative blots). **C** Densitometric quantitative analysis of blot densities (normalised to β-actin) showing significant suppressive effects of siponimod on iNOS upregulation in NMDA treated retinas (****P < 0.0001 one-way ANOVA analysis with Tukey’s multiple comparisons test, *n* = 4 per group). **D** Immunofluorescence images of optic nerve cross sections stained with iNOS (red) and DAPI (blue) (representative images: scale bar, 50 µm; arrows indicate the changes in higher expression of iNOS in NMDA excitotoxicity compared to controls and its decrease with siponimod treatment). **E** Magnified images of optic nerves (representative images, Scale bar: 10 µm) used for quantitative immunoreactivity measurements showing the significant suppressive effects of siponimod treatment on iNOS upregulation during NMDA excitotoxicity (****P < 0.0001, one-way ANOVA analysis with Tukey’s multiple comparisons test *n* = 4 per group)

Nitric oxide plays a crucial role in the induction of oxidative stress and mediating neurotoxic effects in the pathogenesis of several degenerative diseases [49]. The expression of inducible nitric oxide synthase (iNOS) which is induced typically by pro-inflammatory cytokines contributes to large amounts of nitric oxide production that causes neurotoxic effects [50, 51]. We next evaluated the iNOS expression in the retina by immunofluorescence and western blot analysis. Immunofluorescence staining of retinal sections demonstrated increased immunopositivity for iNOS in the GCL and INL in the NMDA-treated group compared to the control mice and the extent of increase was significantly decreased with siponimod treatment (Fig. 7A). Quantitative immunoblot analysis of retinal lysates showed 4.65 ± 0.40-fold upregulation of iNOS in NMDA-treated retinas compared to the control group. Siponimod-treated mice retinas showed significant decrease in iNOS expression (*P* < 0.0001) in NMDA conditions compared to the untreated mice group (Fig. 7B, C). Furthermore, increased immunoreactivity for iNOS in optic nerve cross-sections was increased in response to NMDA excitotoxicity and this increase was reduced significantly (*P* < 0.0001) with siponimod treatment (Fig. 7D, E). These results indicate that siponimod treatment was effective in suppressing iNOS expression in retinal NMAD-induced inflammatory conditions.

### Siponimod Increases Nur77 (NR4A1) Expression to Induce Anti-inflammatory Effects

**Fig. 8:**
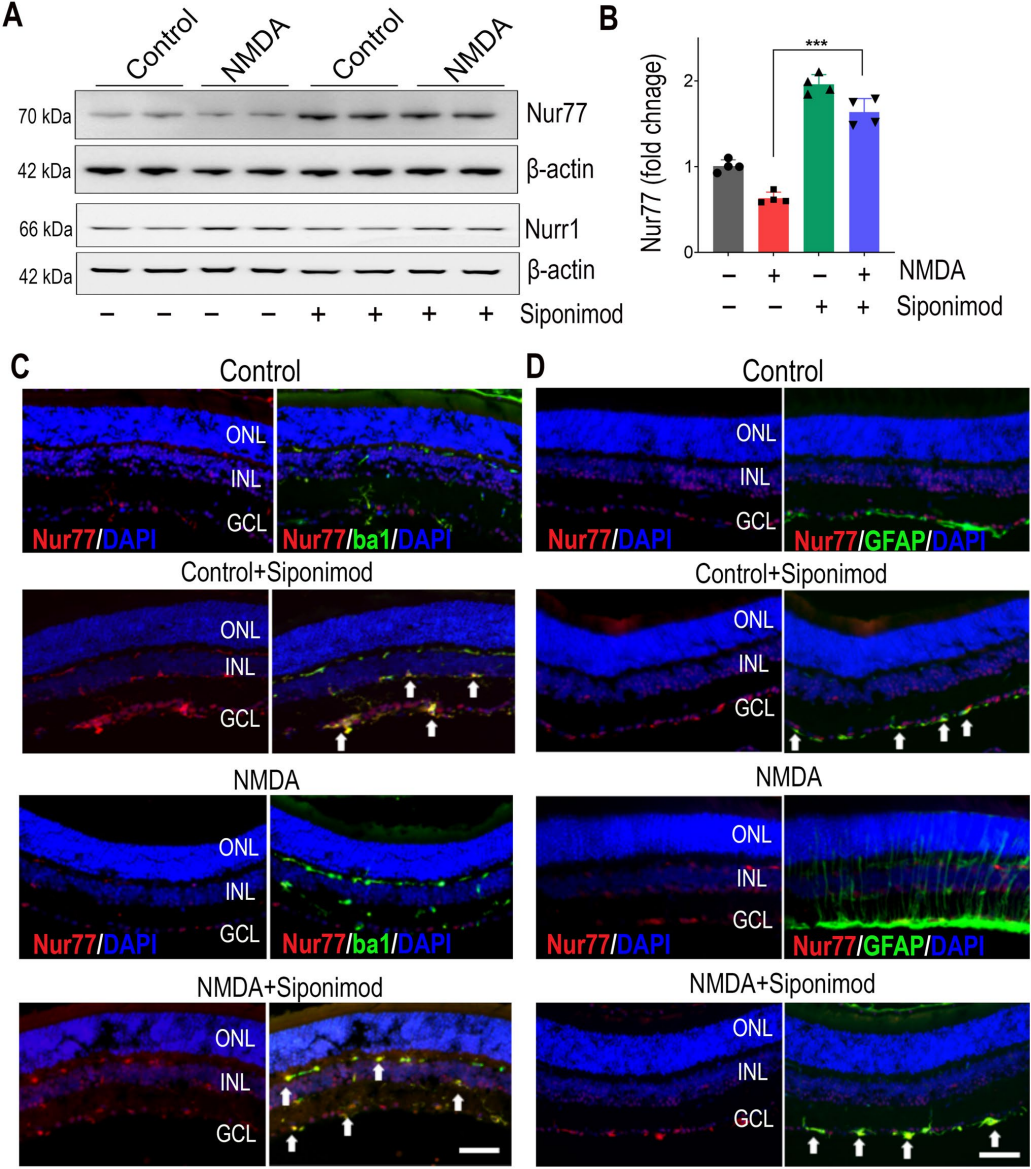
Siponimod treatment increases the expression of Nur77 in retinal NMDA excitotoxicity. Effects of siponimod treatment on expressions of Nur77 and Nurr1 in the retina were evaluated by western blot analysis of retinal tissues following NMDA excitotoxicity. **A** Representative immunoblot bands showing the relative expression levels of Nur77 and Nurr1. **B** Densitometric quantitative analysis of blot densities (normalised to β-actin) showing significant upregulation of Nur77 expression with siponimod treatment during NMDA excitotoxicity (***P < 0.001 one-way ANOVA analysis with Tukey’s multiple comparisons test, *n* = 4 per group). **C** Immunofluorescence images of the eye sections co-stained with Nur77 (red), Iba1 (green), or GFAP (green) and DAPI (blue) (representative images, scale bar: 50 µm; ONL, outer nuclear layer; INL, inner nuclear layer; GCL, ganglion cell layer) showing the induction of Nur77 in mice treated with siponimod (arrows indicate the increased co-labeling of Nur77 with Iba1 and GFAP in siponimod treated mice retinas)

The NR4A orphan nuclear receptor (NR) family of transcription factors has been shown to inhibit inflammatory NF-kB activation [52]. Previously, siponimod treatment increased the expression of NR4A family genes [53]. Among NR4A subfamily receptors, Nur77 (NR4A1) and Nurr1 (NR4A2) expressions in the retina have previously been observed [54, 55]. In NMDA excitotoxicity, we found a significant decrease in Nur77 expression but no changes in Nurr1 expression in western blot analysis of retinal lysates (Fig. 8A). Mice treated with siponimod showed significantly increased Nur77 (2.58 ± 0.15-fold, *P* < 0.001) expression in NMDA-excitotoxicity compared to untreated mice (Fig. 8B). Next, we evaluated the cell-specific induction of Nur77 expression with siponimod treatment by immunofluorescence co-staining of retinal sections with Nur77, Iba1, and GFAP antibodies. In control untreated retinas, Nur77 was stained in the INL and in the GCL along with co-labelled faintly with Iba1 but not with GFAP. The animals treated with siponimod showed markedly increased Nur77 immunoreactivity, and its co-localized was observed with both Iba1 and GFAP in normal and NMDA excitotoxicity conditions (Fig. 8C, D). These results suggest that siponimod treatment increases Nur77 expression in microglia and reactive gliosis to induce the anti-inflammatory effects via the NF-kB-NR4A axis in retinal NMDA excitotoxicity.

## Discussion

In the present study, we investigated in vivo anti-inflammatory effects of the drug siponimod on the retina during NMDA-induced excitotoxicity. Glutamate excitotoxicity through NMDA receptors plays a critical role in glaucomatous retinal degeneration, which is often associated with neuroinflammation via activation of glial cells, inflammatory signaling pathways, inflammasome cascade, and oxidative stress [21, 22, 56, 57]. Our previous study demonstrated the neuroprotective effects of siponimod in experimental glaucoma and in acute NMDA excitotoxicity conditions to protect the inner retinal structure and function [33]. However, the mechanism behind the protective effects of the drug against retinal NMDA excitotoxicity remains unclear. Evidence provided in this study shows that siponimod induces suppression of inflammatory responses during NMDA excitotoxicity which may be responsible for its neuroprotective effects reported previously.

Siponimod is an approved drug prescribed to manage secondary multiple sclerosis and is an S1P structural analogue that selectively modulates S1P1 and S1P5 receptors [31]. The widely accepted mechanism behind the immunomodulatory effect of the drug in MS is via of its effects on S1PRs in the lymphocytes which leads to preventing their egress from secondary lymphoid organs and their infiltration into the CNS[58]. In addition to this effect, siponimod can cross the BBB and the S1PRs expressed by the CNS cells are targets of the drug [32, 59]. Modulation of S1PRs in macroglia cells and microglia during neuroinflammatory conditions has previously been reported to be neuroprotective in various preclinical degenerative models such as chronic CNS inflammatory model of autoimmune encephalomyelitis (EAE), remyelination, mouse model of Huntington’s disease, and neuropathic pain model [60–62]. The activated retinal Müller cells and astrocytes, and microglia, characterized by morphological changes with upregulated inflammatory signaling pathways play a crucial role in various retinal degenerative diseases including glaucoma [7, 8]. Our results showed that glial cells, in both the retina and optic nerve, exhibited morphological changes, proliferation, and upregulation of GFAP in response to NMDA treatment. This activation was significantly reduced in siponimod-treated mice that was evident by immunofluorescence and western blot analysis of the retina and optic nerve. We further investigated the specific inflammatory pathways affected in response to glial cell modulation by siponimod to understand the anti-inflammatory effects during retinal NMDA excitotoxicity.

NF-kB is a key transcriptional regulator of neuroinflammatory signaling and inflammasome activation [63]. The pro-inflammatory cytokines, chemokines, and neurotoxic mediators generated via the NF-kB pathway are known to aggravate neuronal injury[64]. Our results showed significant upregulation of NF-kB p65 expression during NMDA excitotoxicity in both the retina and optic nerve. Siponimod-treated mice retinas demonstrated significantly reduced levels of NF-kB p65 in NMDA excitotoxicity. Suppression of the inflammatory activation of astrocytes and microglia with siponimod via inhibiting the NF-kB pathway and NLRP3 inflammasome has been shown to induce anti-inflammatory effects [31, 62, 65, 66]. Siponimod previously has been shown to increase the expression of NR4A genes which belongs to steroid nuclear hormone receptor family 4, group A [53]. The NR4A subfamily receptors play an important regulatory role in inflammatory responses and have been found to suppress NF-kB pathway activation [67]. Our results showed significant downregulation of Nur77 (NR4A1) in retinal NMDA excitotoxicity. Siponimod treatment significantly increased the Nur77 expression, and this induction was evident in both microglia and reactive gliosis in NMDA excitotoxity. These results suggest that siponimod suppresses the inflammatory activation of microglia and reactive gliosis via NF-kB-NR4A pathway. Inhibition of inflammatory transcription factor NF-kB by NR4A in blood cells and microglia is known to be anti-inflammatory and neuroprotective [52]. We next evaluated the expression of crucial pro-inflammatory cytokines such as TNF-α, IL-1β, and IL-6 to understand the protective effects of siponimod. These pro-inflammatory cytokines are linked with the activated NF-κB signaling pathway and play essential roles in inflammatory-mediated neuronal damage in glaucoma [63, 68]. Our results showed marked upregulation of retinal TNF-α, IL-1β, and IL-6 during NMDA excitotoxicity and this upregulation was significantly suppressed in mice treated with siponimod. Together, these results indicate that siponimod induces anti-inflammatory effects in retinal NMDA excitotoxicity by suppressing NF-kB activation coupled with decreased expression of pro-inflammatory cytokines.

We also identified that siponimod suppresses the NLRP3 inflammasome activation and iNOS expression during retinal NMDA excitotoxicity. NLRP3-IL-1β inflammasome cascade is a critical pathway in neuroinflammatory response and has been implicated in retinal degenerative conditions including glaucoma [46]. NLRP3-inflammasome, a multi-protein complex, mediates cleavage of pro-caspase-1 into mature caspase-1, resulting in IL-1β production [45]. We observed activation of NLRP3 inflammasome with increased levels of cleaved caspase-1 that correlated with matured IL-1β formation in NMDA toxicity. Our results demonstrated that siponimod treatment significantly reduced the NLRP3-IL-1β inflammasome cascade in response retinal NMDA excitotoxicity. Attenuation of NLRP3 inflammasomes via NF-κB pathways in acute glaucoma has been shown to be neuroprotective[69]. In addition, we identified that NMDA excitotoxicity-induced iNOS expression was significantly diminished with siponimod treatment. The expression of iNOS linked with NF-κB signaling is predominantly induced in inflammatory conditions and has been reported to lead to the overproduction of nitric oxide resulting in neuronal destruction via oxidative stress [70, 71]. Siponimod also showed anti-inflammatory and neuroprotective effects both in in vitro and in vivo models of EAE and demyelination [30, 31]. Further, siponimod exhibited significant protective effects in reducing the disability progression in secondary progressive MS patients in a randomized double-blind placebo-controlled phase 3 EXPAND clinical trial [58]. These results suggest that the siponimod drug might directly modulate cells in CNS to induce neuroprotective effects as progressive MS is mainly driven by local CNS degenerative mechanisms[72].

In summary, our study demonstrated that siponimod reduces retinal neuroinflammatory responses during acute NMDA-induced excitotoxicity. Our results provide evidence that the siponimod mediates anti-inflammatory effects by suppressing retinal glial activation coupled with reduced pro-inflammatory cytokines formation and iNOS expression along with inhibition of NF-kB pathway and NLRP3 inflammasome cascade.

## Data Availability

The authors declare that all the relevant data, associated protocols, and materials supporting the findings of this study are present in the paper.
